# Signal or noise? Evaluating commonly used attribution methods for explaining deep neural networks in electrocardiogram classification

**DOI:** 10.1093/ehjdh/ztag038

**Published:** 2026-03-10

**Authors:** Bauke K O Arends, Wouter A C van Amsterdam, Pim van der Harst, Maarten van Smeden, René van Es, Rutger R van de Leur

**Affiliations:** Department of Cardiology, University Medical Center Utrecht, Internal ref E03.511, Heidelberglaan 100, 3584 CX Utrecht, The Netherlands; Department of Data Science and Biostatistics, Julius Center for Health Sciences and Primary Care, University Medical Center Utrecht, Utrecht University, Utrecht, The Netherlands; Department of Cardiology, University Medical Center Utrecht, Internal ref E03.511, Heidelberglaan 100, 3584 CX Utrecht, The Netherlands; Department of Data Science and Biostatistics, Julius Center for Health Sciences and Primary Care, University Medical Center Utrecht, Utrecht University, Utrecht, The Netherlands; Department of Cardiology, University Medical Center Utrecht, Internal ref E03.511, Heidelberglaan 100, 3584 CX Utrecht, The Netherlands; Department of Cardiology, University Medical Center Utrecht, Internal ref E03.511, Heidelberglaan 100, 3584 CX Utrecht, The Netherlands

**Keywords:** Explainable artificial intelligence, Electrocardiogram, Attribution methods, Computer vision, Heatmap

## Abstract

**Aims:**

Attribution-based explainability methods are widely used in electrocardiogram (ECG) analysis to interpret predictions from ‘black-box’ deep neural networks (DNNs). To be useful in clinical applications, attribution methods must produce explanations that are both clear and reflective of the model’s inner workings. This study evaluates 12 attribution methods in DNN-based ECG classification.

**Methods and results:**

We analysed 12 attribution methods using a dataset of 873 710 median beat ECGs spanning nine diagnostic classes. Methods were applied to convolutional neural network-based models trained for ECG classification. Performance was evaluated across four experiments: inter-method similarity, self-consistency, dependence on model weights, and ability to identify features important for model inference. All task models achieved an area under the receiver operating curve above 0.95. Attribution methods demonstrated low correlation and high variability across inter-method comparisons. Self-consistency across random model initializations was moderate for most methods (mean correlation 0.41–0.65). Randomizing model weights led to rapid loss of correlation, although some methods did not converge to zero. Perturbation of input data revealed differences in how well attribution methods identified features relevant to model performance.

**Conclusion:**

Attribution methods demonstrated limited reliability, instability across model variants and incomplete dependence on learned parameters, constraining their utility in high-stakes settings such as healthcare. These findings suggest that attribution techniques should be used cautiously and supported by task-specific sanity checks. Approaches grounded in rigorous validation, inherently interpretable modelling or counterfactual explanations may better support clinically meaningful insight.

## Graphical Abstract

**Graphical Abstract:**
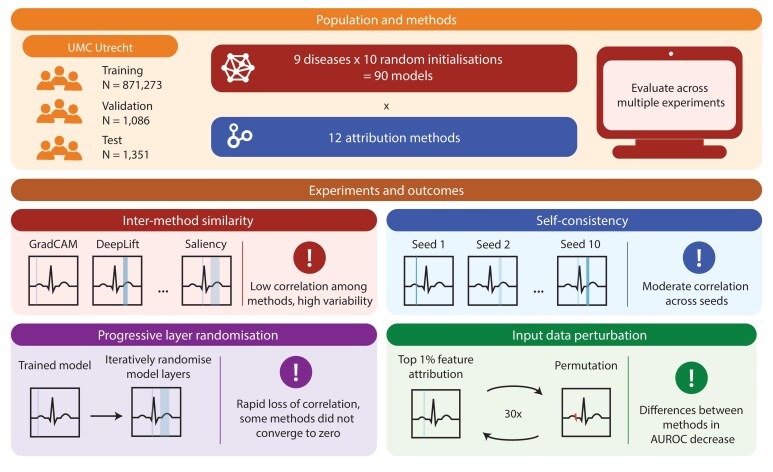

## Introduction

Deep neural networks (DNNs), a subset of artificial intelligence (AI) algorithms, have demonstrated strong performance in automating the interpretation of electrocardiograms (ECGs), assisting physicians in tasks such as rhythm analysis and identifying abnormalities that may be challenging to detect by eye, such as reduced ejection fraction.^1–3^ However, despite their potential benefits, the ‘black-box’ nature of many DNN algorithms, where the relationship between input and output remains opaque, may pose a challenge for clinical adoption.^4^

To address this lack of transparency, explainable AI (XAI) methods have emerged to offer post hoc insight into model behaviour. Among the most widely used are attribution-based methods, which aim to quantify the importance of each input data point, such as a time point in an ECG, to a model’s prediction.^5,6^ In ECG classification, these methods produce heatmaps (or attribution maps) that highlight the segments of the ECG the model is believed to consider important. Although attribution methods are routinely used in clinical AI research, they are rarely evaluated beyond small sets of illustrative examples.^7^ This practice assumes that explanations that ‘look right’ are also trustworthy, an assumption that is increasingly being challenged.

Evidence from image classification studies has shown that some attribution methods yield nearly identical heatmaps even when the model weights are randomized,^8,9^ or produce different explanations for the same input depending on the chosen method.^8–10^ These findings suggest that attribution methods may not reflect the model’s true reasoning, and may create a false sense of interpretability. While one-dimensional signals like ECGs have received less scrutiny, preliminary studies indicate similar concerns, with attribution maps varying significantly across methods and model instances.^11–13^

To be trustworthy, attribution methods should provide explanations that are both interpretable and faithful to the model’s inner workings. Markus *et al*. define two key criteria for this: Interpretability, meaning that the explanation is clear (pointing to consistent features) and parsimonious (not overly complex); and fidelity, meaning that the explanation truthfully reflects how the model arrives at its prediction, encompassing completeness (sufficiently providing information to compute the output for a given input) and soundness (accurately reflecting the model’s decision-making process).^14^

However, many attribution methods in use today have not been rigorously tested against these criteria. Despite their growing role in clinical AI, few studies have evaluated whether these methods are interpretable or faithful in practice. This is particularly problematic because attribution methods are increasingly used to validate whether models rely on clinically meaningful features (e.g. the PR interval in AV block), or to explore novel features in diseases that are not yet well understood. If the methods themselves are unreliable, the downstream conclusions drawn from them, whether scientific, clinical, or regulatory, may be flawed.

In this study, we evaluate 12 widely used attribution methods in the context of deep learning-based ECG classification. Using a set of four experiments, we assess: (1) similarity across methods, (2) consistency across model initializations, (3) the ability of attribution methods to highlight diagnostically important features, and (4) whether the resulting maps are dependent on the model’s learned parameters. Through this evaluation, we aim to provide an assessment of the clarity and soundness of these methods, and to support the responsible and informed use of XAI in clinical AI research.

## Methods

### Study population

All resting 12-lead 10-s ECGs, sampled at 250 and 500 Hz and recorded between 1997 and 2020, were extracted from the University Medical Center Utrecht digital archive. The extracted data were deidentified in accordance with the European Union General Data Protection Regulation and written informed consent was therefore not required by the University Medical Center Utrecht ethics committee. Patients under the age of 16 were excluded. This large, real-world clinical dataset spanning a broad range of diagnoses offered a stable and representative foundation for examining attribution behaviour independent of idiosyncrasies of smaller or more selective cohorts.

### ECG processing and outcome selection

ECGs recorded at 250 Hz were resampled to 500 Hz using linear interpolation. The median beat ECG was generated at the time of acquisition by the contemporaneous Marquette 12SL algorithm (MUSE; GE Healthcare), which aligns beats within the 10-s recording and computes a representative, noise-reduced cycle. This approach avoided the beat-to-beat variability present in full-length recordings that would introduce unwanted heterogeneity into the attribution analyses.

All ECGs were interpreted by the same Marquette 12SL algorithm used at acquisition or, when available, corrected by a physician. Free-text ECG interpretations were converted to structured data using a text mining-based approach described before.^15^ Nine diagnostic ECG classes were selected based on data availability (e.g. representing >3% of the total dataset) and ease of automatic classification. The selected classes were sinus rhythm, atrial fibrillation, sinus bradycardia, sinus tachycardia, first-degree atrioventricular (AV) block, right bundle branch block, left bundle branch block, low QRS voltage, and left ventricular hypertrophy.

### Model architecture and algorithm development

We employed a convolutional neural network using a ResNet architecture described before,^16^ consisting of a total of 22 one-dimensional convolutional layers with a kernel size of seven that doubled the channel dimension and halved the spatial dimension every two layers. All convolutional layers were followed by batch normalization modules, dropout and rectified linear unit activation functions. For every ECG, eight leads (I, II, V1–V6) were used as input for the model. An overview of the model architecture and parameters is provided in Supplementary material online, *Table S1*. We selected this architecture because it is widely used in ECG deep learning, offers stable and well-characterized performance, and provides a representative convolutional structure for evaluating attribution behaviour without introducing architectural confounders.

Data were split at the patient level to ensure no overlap between the training and test sets. A validation set was created by randomly selecting 250 patients (1086 ECGs), and an independent test set was created by selecting another 250 patients (1351 ECGs). All remaining ECGs were used for training. This split provided sufficient representation of all diagnostic classes in both the validation and test sets while keeping these sets small enough to enable the large-scale attribution analyses that form the core of this study. The computational burden of this work arises primarily from the generation of attribution maps rather than from model training or inference. Each attribution map requires repeated gradient-based or sampling-intensive operations, making attribution analysis several orders of magnitude more demanding than forward-pass model evaluation. Restricting the size of the validation and test sets therefore allowed the attribution experiments to be conducted without compromising representation across diagnostic categories. The model was trained as a binary classifier for each diagnostic class, with ten training repetitions using different random initializations of the model weights, resulting in a total of 90 distinct models. All models and analyses were implemented using Python 3.8.10 and PyTorch, running on an NVIDIA A6000 GPU (48 GB RAM).

### Attribution methods

We compared a total of 12 popular attribution methods in the context of ECG classification, including Saliency,^17^ SmoothGrad,^18^ Gradient*Input,^19^ Guided backpropagation,^20^ GradCAM,^21,22^ Guided GradCAM,^21^ Shapley sampling,^23^ DeepLift,^19^ Integrated gradients (IG),^24^ IG + SmoothGrad,^18,24^ GradientSHAP,^23^ and DeepLiftSHAP.^23^ For attribution methods requiring parameter tuning, hyperparameters influencing output quality (e.g. number of segments, number of samples and approximation steps) were adjusted iteratively until further changes had no visible effect on the output. While this is a commonly applied heuristic, objective criteria for hyperparameter selection remain an open research question. The final parameter settings for all methods are provided in Supplementary methods. We used the implementations of all attribution methods from the Captum package.

### Statistical analysis

The discriminatory performance of the algorithms was assessed using the mean values of area under the receiver operating characteristic curve (AUROC), area under the precision recall curve (AUPRC), precision, recall, accuracy, micro F1 score, and specificity across algorithm seeds. The optimal probability threshold for classification was identified using Youden’s *J* statistic on the validation set.

**Figure 1:**
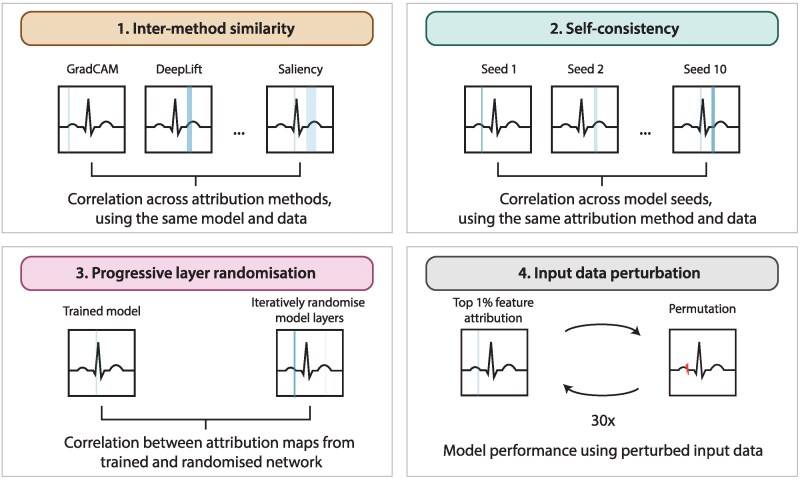
Examples of the four experiments conducted. Each panel illustrates one of the four experiments designed to evaluate the clarity and soundness of attribution methods. (1) Inter-method similarity compares attribution maps generated by different attribution techniques applied to the same model and ECG input. (2) Self-consistency assesses the reproducibility of attribution maps across models trained with different random seeds but on the same data. (3) Progressive layer randomization evaluates how attribution maps change as layers of a trained model are incrementally randomized, testing dependence on model weights. (4) Input data perturbation quantifies the impact on model performance when top-ranked input features, according to each attribution method, are iteratively perturbed.

**Figure 2:**
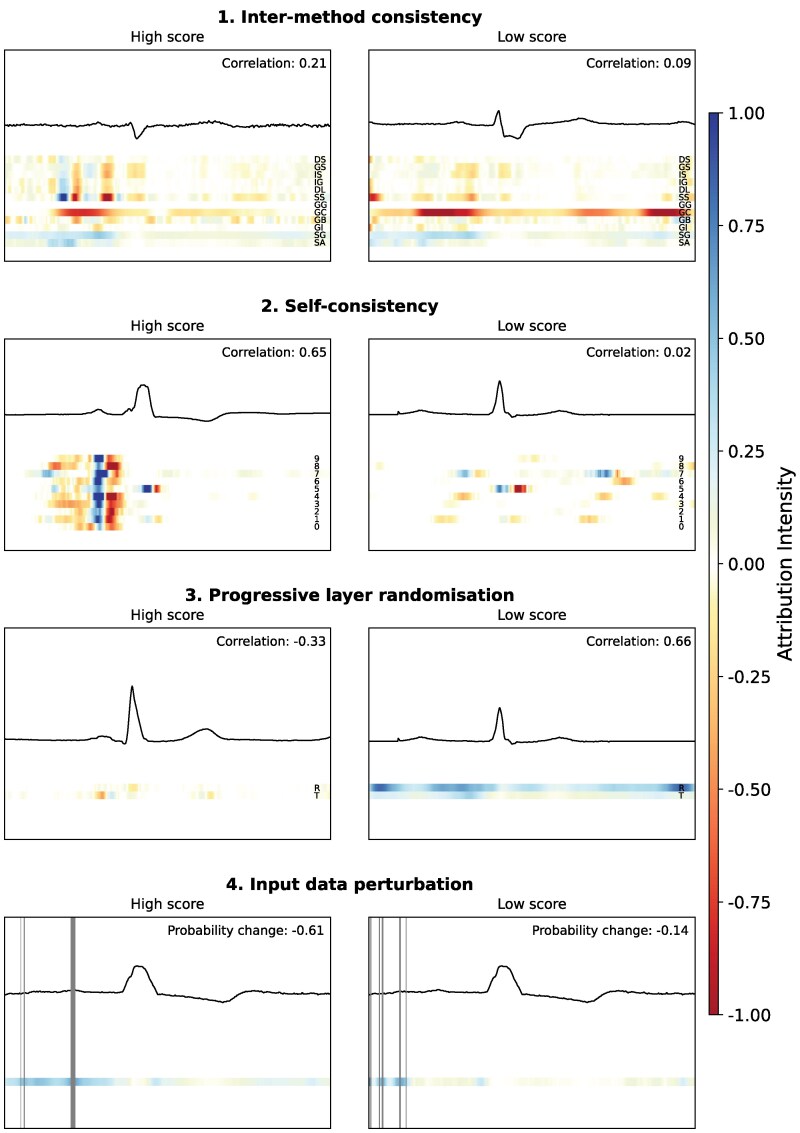
Examples of the four experiments conducted, each illustrating one example with a high score and one with a low score. Each coloured line represents a separate attribution plot, with the mean pair-wise correlation or predicted probability change displayed in the top right corner. The second experiment examples use Guided GradCAM as attribution method. The third experiment uses Guided GradCAM for the first example, and SmoothGrad for the second example. The fourth experiment displays Integrated Gradients for the first example, and SmoothGrad for the second example. Abbreviations: DS, DeepLiftSHAP; GS, GradientSHAP; IS, IG SmoothGrad; IG, Integrated Gradients; DL, DeepLift; SS, Shapley sampling; GG, Guided GradCAM; GC, GradCAM; GB, Guided Backpropagation; GI, Gradient*Input; SG, SmoothGrad; SA, Saliency; 0–9, seed number; R, randomized; T, unrandomized.

To characterize the diversity of attribution methods, attribution maps were first smoothed using a Savitzky–Golay filter (31-sample window, third-order polynomial) to reduce high-frequency gradient noise and stabilize peak locations while preserving overall waveform morphology. Pearson correlation coefficients ρ were then computed between pairs of attribution maps for each ECG. Correlations were calculated pointwise across all time samples and leads and then averaged to obtain a single similarity score per ECG (*Figures 1* and *2*). These scores were then aggregated across different model seeds and diagnostic classes to determine overall inter-method similarity. We used Pearson correlation because attribution maps often exhibit continuous, small-scale variations in magnitude that reflect model-specific sensitivity to input features, and our aim was to assess linear similarity in both shape and amplitude rather than monotonic correspondence alone. For completeness, and to assess robustness to outliers and monotonic transformations, we also report Spearman correlations in Supplementary Materials. Because the aim of this analysis was to describe how attribution methods relate to one another rather than to evaluate their correctness, correlation was interpreted as an index of methodological similarity rather than a measure of faithfulness.

Three subsequent experiments were conducted to evaluate the soundness and clarity of attribution methods (*Figures 1* and *2*). The first experiment assessed self-consistency by measuring the correlation of attribution maps across different model initializations. Pearson correlation coefficients were calculated between attribution maps generated from models trained with different random seeds but on the same data. These correlations were averaged across diagnostic classes to obtain an overall measure of self-consistency.

The second experiment evaluated the dependence of attribution maps on the model’s learned parameters. To test this, model weights were progressively randomized layer by layer, starting from the output layer and moving towards the input layer. After each randomization step, new attribution maps were generated and their Pearson correlation with the original maps was computed. To reduce computational burden, the progressive randomization was performed on a single model trained to detect atrial fibrillation.

The third experiment assessed the ability of attribution methods to highlight meaningful features by perturbing highly attributed regions in the input ECG and measuring the effect on model performance. A modified version of the ‘remove and debias’ approach was applied, which mitigates class information leakage more effectively than traditional removal-based methods such as Most Relevant First (MoRF) and Least Relevant First (LeRF).^25^ Instead of completely removing data points, the top 1% of most highly attributed data points were iteratively replaced in 30 steps, resulting in a total of 30% of the input data being perturbed. Perturbed values were generated using linearly interpolated neighbouring data points while introducing a small amount of Gaussian noise (*σ* = 0.25) to prevent the model from learning a simple linear relationship. Model inference was rerun after each perturbation step, and performance was reassessed to evaluate how rapidly classification accuracy degraded as increasingly important regions were perturbed. The choice of 30% perturbation was based on empirical testing, balancing a meaningful reduction in model performance while ensuring that the perturbed input still retained enough information for interpretation.

## Results

### Data characteristics and discriminatory performance of DNN algorithms

A total of 873 710 ECGs from 192 257 individual patients (45.1% female, mean age 60.9 years) were included in the analysis (*Table 1*). Sinus rhythm was the most prevalent diagnostic class (62.9%), while low QRS voltage was the least common (3.6%). All models achieved a high discriminatory performance with a mean AUPRC above 0.85 for eight out of nine classes, with the exception of sinus bradycardia which had a mean AUPRC of 0.64. Mean AUROC values exceeded 0.95 for all classes. Full model performance metrics are available in Supplementary material online, *Table S2*.

**Table 1:** Composition of train, validation, and test sets used to train the binary classification models

	Train	Validation	Test
ECG, *n*	871 273	1086	1351
Patients, *n*	191 757	250	250
No. ECGs per patient, Mean (SD)	4.5 (8.6)	4.3 (6.7)	5.4 (11.4)
Female patients, *n* (%)	86 537 (45.1)	101 (40.4)	119 (47.6)
Age, Mean (SD)	60.9 (15.4)	61.0 (15.4)	64.3 (13.7)
Sinus rhythm, *n* (%)	547 877 (62.9)	740 (68.1)	804 (59.5)
Atrial fibrillation, *n* (%)	53 872 (6.2)	48 (4.4)	88 (6.5)
Sinus bradycardia, *n* (%)	93 710 (10.8)	101 (9.3)	143 (10.6)
Sinus tachycardia, *n* (%)	62 338 (7.2)	73 (6.7)	112 (8.3)
First degree AV block, *n* (%)	53 320 (6.1)	69 (6.4)	86 (6.4)
Right bundle branch block, *n* (%)	72 057 (8.3)	94 (8.7)	239 (17.7)
Left bundle branch block, *n* (%)	36 950 (4.2)	53 (4.9)	115 (8.5)
Low QRS voltage, *n* (%)	31 056 (3.6)	41 (3.8)	37 (2.7)
Left ventricular hypertrophy, *n* (%)	61 548 (7.1)	103 (9.5)	104 (7.7)

The same ECGs were used for each model, only the class labels were varied.

Abbreviations: AV, atrioventricular; ECG, electrocardiogram; SD, standard deviation.

### Cross-method similarity of attribution methods

**Figure 3:**
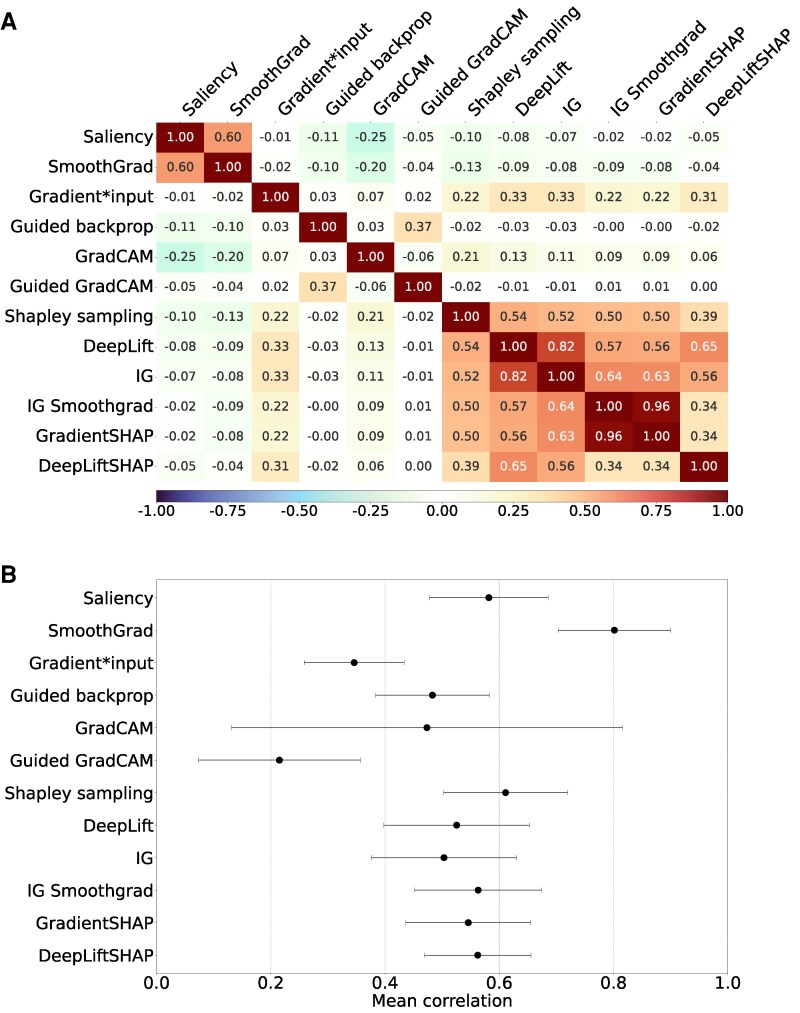
(*A*) Inter-method similarity: Pearson correlation between attribution maps produced using different attribution methods, for the same model (seed) and data. Results were aggregated over nine classes and ten seeds. (*B*) Self-consistency: Pearson correlations between attribution maps across seeds produced by the same attribution method, averaged over ten seeds and nine classes. Abbreviations: IG, integrated gradients.

Across the 12 attribution methods, the Pearson correlation structure showed partial clustering that was consistent with the methods’ underlying mathematical principles (*Figure 3A*, Supplementary material online, *Figure S1*). Saliency and SmoothGrad showed the highest agreement among the sensitivity-based techniques, consistent with their shared reliance on local gradients. Gradient*Input, although also gradient-based, did not correlate with these methods and instead showed moderate correlation with the baseline-dependent feature-contribution methods, which rely on integrating gradients relative to a reference input. Within this baseline-dependent group, correlations ranged from 0.34 for DeepLiftSHAP to 0.96 for the closely related IG SmoothGrad and GradientSHAP, indicating a generally coherent set of attribution behaviours. Activation-based methods produced distinct and comparatively unstable patterns, with correlations *ρ* < 0.10 in this group. The only notable correspondence was the correlation of 0.37 between Guided backpropagation Guided Grad-CAM, which also exhibited high variability across seeds. Correlations across method families were uniformly low, reflecting the differing assumptions and interpretive objectives that underlie these attribution techniques. Similar results were observed using Spearman correlation (see Supplementary material online, *Figure S2*).

### Self-consistency of attribution methods

If attribution methods provide stable and reliable explanations, they should produce similar attribution maps across models trained on the same data but with different random initializations. However, we observed substantial variation in self-consistency, with mean correlation between attribution maps across different model initializations ranging from 0.41 to 0.65. Guided GradCAM showed the lowest self-consistency (*ρ* = 0.29 ± 0.28), while SmoothGrad showed the highest (*ρ* = 0.82 ± 0.12) (*Figure 3B*). The highest variability was observed for GradCAM (standard deviation 0.39). Across diagnostic classes, mean correlation over all methods ranged from 0.54 (low QRS voltage, left ventricular hypertrophy) to 0.62 (first-degree AV block). Supplementary material online, *Table S3*, Supplementary material online, *Figures S3* and *S4* provide a detailed overview of all self-consistency results.

### Dependence on model weights

**Figure 4:**
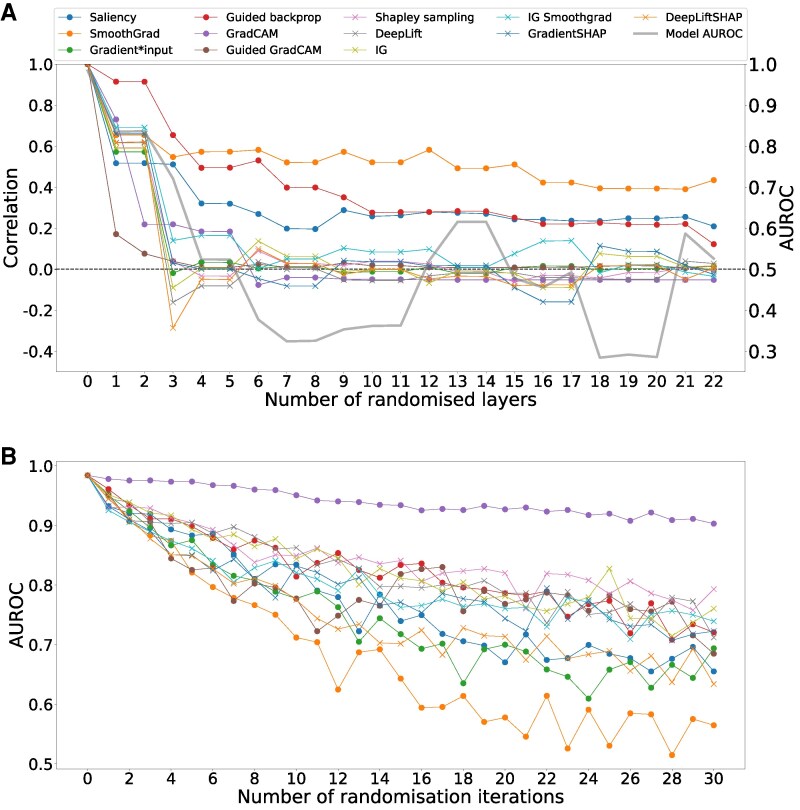
(*A*) Dependence on model weights: +progressive randomization of a model trained to detect atrial fibrillation. The *y*-axis shows the Pearson correlation between the attribution maps produced using the non-randomized model and each randomization step. The *x*-axis shows the amount of randomized convolutional layers, starting at the output layer of the network. (*B*) Ability to identify features important to model prediction: iterative perturbation of 1% of input data based on the highest absolute attribution values and the resulting model performance. The original data points are replaced with a linear imputation of the neighbouring data points, multiplied with Gaussian noise.

If attribution methods reflect model reasoning, we would expect attribution maps to lose coherence when model weights are randomized. As anticipated, correlation between original attribution maps and those generated after randomizing model weights declined for all methods within the first three randomization steps, at the same rate as model AUROC (*Figure 4A*). Most methods converged to near-zero correlation, except for Saliency, SmoothGrad, and Guided Backpropagation, which exhibited a slower decline and did not fully reach zero correlation after complete model randomization. Similar results were observed using Spearman correlation (see Supplementary material online, *Figure S5*).

### Perturbing input data

If attribution maps accurately highlight features essential to model prediction, perturbing these features should cause a significant decline in model performance. We found that perturbing features identified by SmoothGrad, DeepLiftSHAP, Gradient*Input, and Saliency led to the most rapid performance degradation, with AUROC dropping to approximately 0.50 for SmoothGrad and 0.65 for the other three methods after 30% of the input data was perturbed (*Figure 4B*). Most other attribution methods showed a similar pattern of performance degradation, converging to an AUROC of 0.75. Perturbation of features highlighted by GradCAM led to a more gradual decline of model performance with a final AUROC of 0.92.

## Discussion

Attribution methods are widely used in clinical AI research to gain insight into how DNNs arrive at their predictions. Yet the extent to which these methods offer a reliable or coherent view of model behaviour remains uncertain. In this study, we applied 12 widely used attribution techniques to 90 CNNs trained on ECG classification tasks, generating more than 1.5 million attribution maps to characterize their behaviour in a systematic manner. The methods produced distinct attribution patterns, reflecting the different quantities they are designed to measure, and this diversity shows that attribution techniques cannot be treated as interchangeable. The remaining experiments therefore focused on properties that extend beyond these inherent methodological differences. Stability across random initializations, behaviour under progressive model randomization and sensitivity to perturbation of highly attributed regions each provided further insight into how consistently these methods relate to the model’s predictions. Across these analyses, several methods showed limited stability, weak correspondence with model reasoning or insufficient sensitivity to features that influenced the model’s output. These findings indicate that the explanatory value of current attribution techniques is constrained and highlight the need for careful methodological choice and interpretation when explainability is used to support clinical trust, model validation or regulatory assessment.

### Attribution methods are not faithful to model behaviour

Our inter-method similarity experiment showed that attribution techniques produced distinct patterns of relevance, with partial clustering according to their mathematical foundations. Sensitivity-based approaches, such as saliency and SmoothGrad, emphasized local gradients and therefore highlighted sharp changes in model response. Gradient*Input altered this pattern by weighting gradients by the amplitude of the ECG signal, which shifted its relevance towards prominent morphological deflections and resulted in moderate similarity to baseline-dependent feature contribution methods. These contribution methods, including Integrated Gradients, DeepLIFT and their variants, compare the input with a reference and generally show consistent internal agreement. However, not all methods behaved consistently within their respective families. The class activation mapping-based techniques demonstrated low internal similarity, and DeepLiftSHAP showed weaker correspondence with closely related methods, suggesting that these approaches may be less stable or less well suited to the structure of one-dimensional ECG data. Together, these findings illustrate that the interpretive behaviour of attribution methods is shaped by both their mathematical assumptions and their interaction with ECG morphology, and underscore the importance of choosing methods whose properties align with the characteristics of the data.

We further tested model dependency by progressively randomizing model weights. If attribution methods are truly reflective of the model’s learned reasoning, randomization should change their outputs. As expected, most methods did show reduced correlation after randomization, in line with the model’s performance degradation. However, Saliency, SmoothGrad, and Guided Backpropagation failed to converge to zero correlation, suggesting that their attributions are not fully dependent on the model’s learned parameters. In practice, this raises the risk that clinicians might rely on heatmaps that are not tied to the actual prediction logic of the model.

To evaluate whether attribution methods identify clinically important features, we perturbed ECG segments that were ranked as most important by each method. Performance dropped most steeply for SmoothGrad, DeepLiftSHAP, Input*Gradient, and Saliency. In contrast, GradCAM led to minimal change in model performance, suggesting a weaker connection between its attribution outputs and true model reliance. In a clinical workflow, this discrepancy could lead to misplaced trust in irrelevant regions of the ECG signal.

### Attribution methods lack clarity and consistency

For attribution methods to be useful in practice, they must offer clear and consistent explanations.^14^ However, we observed only moderate self-consistency across models trained with different random seeds on the same data and task (*ρ* = 0.52 ± 0.14). According to the implementation invariance axiom, functionally equivalent models should yield similar attributions.^24^ The models in our study all achieved high AUPRC values, yet attribution maps varied considerably. This likely stems from the non-convexity of neural network optimization, where different initializations can lead to different, but equally valid, internal representations. While this may be acceptable from a performance standpoint, it raises doubts about using attribution maps for robust feature discovery. This issue becomes even more important in simple classification tasks such as sinus rhythm prediction, where only a limited number of relevant segments are expected. Yet, even in this case, attribution map correlation between seeds remained low (*ρ* = 0.57 ± 0.13), highlighting the lack of consistency.

Beyond consistency, attribution methods suffer from limited interpretability. Heatmaps show that ‘something important’ lies in a highlighted region but offer no specificity. For instance, highlighting the T-wave could point to the QT interval, T-wave height, or morphology.^4^ This ambiguity limits clinical interpretability. Attribution methods also implicitly assume that each feature contributes independently to a prediction, which oversimplifies clinical reasoning. Many diagnoses rely on feature interactions. For example, ST-segment elevation in V2–V3 is not inherently pathological but becomes diagnostically relevant when accompanied by reciprocal changes or Q-waves, indicating ischaemia. These limitations are also addressed in prior work showing that attribution maps often fail to match expert-defined regions.^8,12^ Furthermore, clinicians have rated such maps as only marginally helpful (average score 0.66 on a −3 to +3 scale).^26^ When used locally (i.e. for individual predictions), attribution methods are also prone to confirmation bias, especially if only favourable examples are cherry picked. While global attribution methods, like Shapley values or global aggregation of local attribution methods, offer more robust insights, they are computationally expensive and still affected by underlying assumptions.^27^

Several recent studies have also examined attribution behaviour in ECG models, providing valuable context for our findings. Storås *et al*. evaluated multiple gradient-based attribution methods for predicting ECG amplitudes and intervals and showed that attribution patterns can vary substantially between techniques and across tasks, while Goettling *et al*. used perturbation analyses to examine model reliance on specific ECG segments and similarly found that class activation mapping approaches often performed poorly.^12,28^ Our results align with these observations, as class activation mapping-based methods also showed weak correspondence with model behaviour in our experiments and attribution performance varied considerably across diagnostic classes and tasks. The present study extends this prior work by evaluating a broader collection of attribution techniques across nine real-world diagnostic tasks, multiple independently trained models and several complementary experiments. This wider scope reveals systematic instability within attribution methods and highlights how the interaction between method assumptions and ECG morphology contributes to divergent explanatory patterns. Through this comprehensive evaluation, our findings provide a more detailed characterization of attribution behaviour in ECG-based deep learning and highlight key challenges that may limit their clinical applicability.

To place these observations into a broader conceptual framework, we propose six levels of explainability that distinguish between increasingly meaningful forms of insight (*Table 2*). Attribution maps occupy level 1 of this hierarchy, offering coarse localization without specifying feature identity, diagnostic meaning or causal reasoning. Higher levels require understanding of how predictions change with specific input modifications, the recognition of clinically interpretable features and the integration of multiple elements in a manner that resembles expert reasoning. Recent advances in counterfactual explainability offer a potential path towards levels 2 and 3. Generative counterfactual methods create alternative versions of the input that shift model predictions and can reveal the kind of morphological changes the model relies upon.^29^ Such approaches, including those based on variational autoencoders and diffusion models, have begun to produce clinically recognizable modifications such as altered T-wave morphology or PR-interval changes in ECGs.^30^ Prototype-based models provide a complementary route towards these levels by explaining predictions through reference to learned ECGs rather than through synthetic input modification.^31^ Although these techniques are often described collectively as explainability methods, they differ in important ways. Classical counterfactuals provide local, instance-specific modifications that show how a single ECG must change to affect the model’s prediction, whereas latent traversals in generative models move along smooth directions in a learned representation space and therefore reveal broader trends in the data. Neither approach yields a global causal rule or direct control over predicted probabilities, but together they illustrate how explanation can move beyond coarse localization towards feature-level meaning. Their reliability, however, depends on the quality of the generative or prototype model and the assumptions implicit in the construction of the explanation.

**Table 2:** Proposed framework for levels of explainability in electrocardiogram-based artificial intelligence

Level	Name	Description	Example
0	No explainability	The model outputs a prediction without any indication of reasoning.	Atrial fibrillation: 97%
1	Localization	The model highlights which part of the ECG was important to the prediction.	Heatmap over *P*-wave region in V1
2	Feature modulation	The model shows how changes in the ECG would affect its prediction.	Artificial shortening of the PR interval increases confidence in sinus rhythm.
3	Named features	The model assigns clinical terms to the important region.	ST-segment elevation in V2
4	Causal reasoning	The model explains why the feature supports the diagnosis.	Inferior ST elevation with reciprocal changes suggests myocardial infarction
5	Expert-level explanation	The model mimics an expert. Integrates features, context, and evidence.	Ischaemia likely due to ST elevation + prior history; consistent with literature. Without intervention, Q-waves will likely develop within the next hours.

### Recommendations for practice and further research

The findings of this study have important implications for clinical and regulatory practice. Attribution maps are frequently invoked as evidence that a model behaves sensibly or attends to clinically relevant features, yet our analyses show that they can be inconsistent, ambiguous and insufficiently tied to model reasoning. Their performance is also highly task dependent, and there is currently no agreed method to determine which attribution method, if any, is appropriate for a given prediction problem.^11,13,32^ In safety-critical environments, such limitations can create misplaced trust and obscure failure modes. More robust approaches, including external validation across diverse populations, inherently interpretable models or richer explanatory frameworks such as counterfactual and concept-based methods, may offer more actionable insight.^11,14,30^ Where attribution techniques continue to be used, they should be accompanied by clear justification, task-specific validation and careful interpretation.

### Strengths and limitations

A major strength of our study is its comprehensive scope. We evaluated 12 attribution methods across nine classification tasks and 90 model initializations, generating over 1.5 million attribution maps. This large-scale analysis allowed for robust, comparative testing of key explainability criteria. However, we did not evaluate all existing attribution techniques or hyperparameter setting due to computational constraints. Nevertheless, the included methods are among the most commonly used, and the consistency of our findings suggests that the conclusions are unlikely to change with additional methods.^5,6^ Model performance could theoretically influence attribution reliability, particularly for rare classes, but we observed similar attribution behaviour in tasks with higher and lower AUPRC values. This indicates that the conclusions predominantly reflect properties of the attribution methods rather than differences in classifier accuracy. We also did not implement generative counterfactual methods, which limits empirical comparison with alternative forms of explanation. Future work should evaluate these approaches alongside attribution techniques to clarify which methods produce clinically meaningful insight across different tasks and model architectures.

## Conclusion

This study highlights fundamental limitations in the reliability of attribution methods for DNNs applied to ECG interpretation. Despite extensive testing, these methods fail to meet key XAI criteria, demonstrating high variability across tasks, methods, and model initializations. Their task-dependent nature and lack of consistency make them unsuitable for critical applications such as healthcare, where explainability is important for bias detection and model trustworthiness. We therefore recommend caution in using attribution methods and advocate for alternative explainability approaches that provide more robust, reproducible, and clinically meaningful insights. If attribution methods are still used, they should be treated as exploratory tools rather than definitive explanations. Sanity checks, task-specific validation, and transparent justification of method choice are essential to ensure their responsible use in clinical AI research.

## Lead author biography


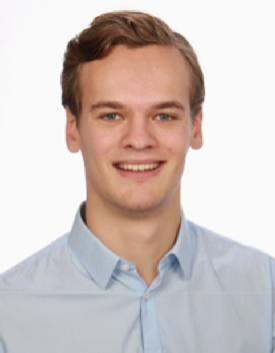


Bauke K.O. Arends is a physician-scientist specializing in the intersection of electrocardiography and deep learning. His research focuses on developing and validating AI-ECG algorithms to streamline diagnostic workflows and improve the detection of cardiovascular disease. He is particularly interested in advancing explainable and generalizable deep learning methods to enable safe and effective clinical integration of AI in cardiology.

## Supplementary Material

ztag038_Supplementary_Data

## Data Availability

Data used to train the algorithms in this study are not available due to privacy concerns. Code used to train the models and run all analyses is publicly available through < URL AVAILABLE UPON PUBLICATION > .
