# Supplementary material for: Signal or noise? Evaluating commonly used attribution methods for explaining deep neural networks in electrocardiogram classification
Source: Eur Heart J Digit Health. 2026 Mar 10;7(3):ztag038. doi: 10.1093/ehjdh/ztag038 (PMC12980500; doi:10.1093/ehjdh/ztag038)
Supplement: ztag038_Supplementary_Data [file ztag038_supplementary_data.docx]

**Supplementary materials**

Table of contents

[Supplementary methods 2](#_Toc209465193)

[Supplementary figures 3](#_Toc209465194)

[Supplementary tables 8](#_Toc209465195)

# Supplementary methods

*Attribution method hyperparameter settings*

The methods Saliency, Gradient*input, Guided backpropagation, GradCAM and Guided GradCAM have no additional configuration options and were used as is. For Shapley sampling, DeepLift, Integrated gradients (IG) and GradientSHAP, the ‘baselines’ argument was set to the mean electrocardiogram (ECG) of the validation set. Shapley sampling was configured to use a mask consisting of 240 segments, equivalent to 30 segments per lead with a width of 20 samples at 500 Hz. For IG and IG SmoothGrad, the number of approximation steps was set to 64. The SmoothGrad and IG + SmoothGrad both used a standard deviation of 0.1.

For each method that involves a sampling operation (SmoothGrad, Shapley Sampling, IG + SmoothGrad and GradientSHAP), the number of samples was set to ensure a minimum correlation of 0.98 between the attribution maps produced when using the same input sample and model. We take this approach to correct for the natural decrease in correlation caused by the sampling operations. Finetuning the settings based in this criterion yielded 128, 128, 64 and 256 samples for SmoothGrad, Shapley Sampling, IG + SmoothGrad and GradientSHAP, respectively. Similarly, for DeepLiftSHAP baselines were randomly sampled from the validation set, with 128 baselines determined to be the optimal sample size.

Supplementary figures

**
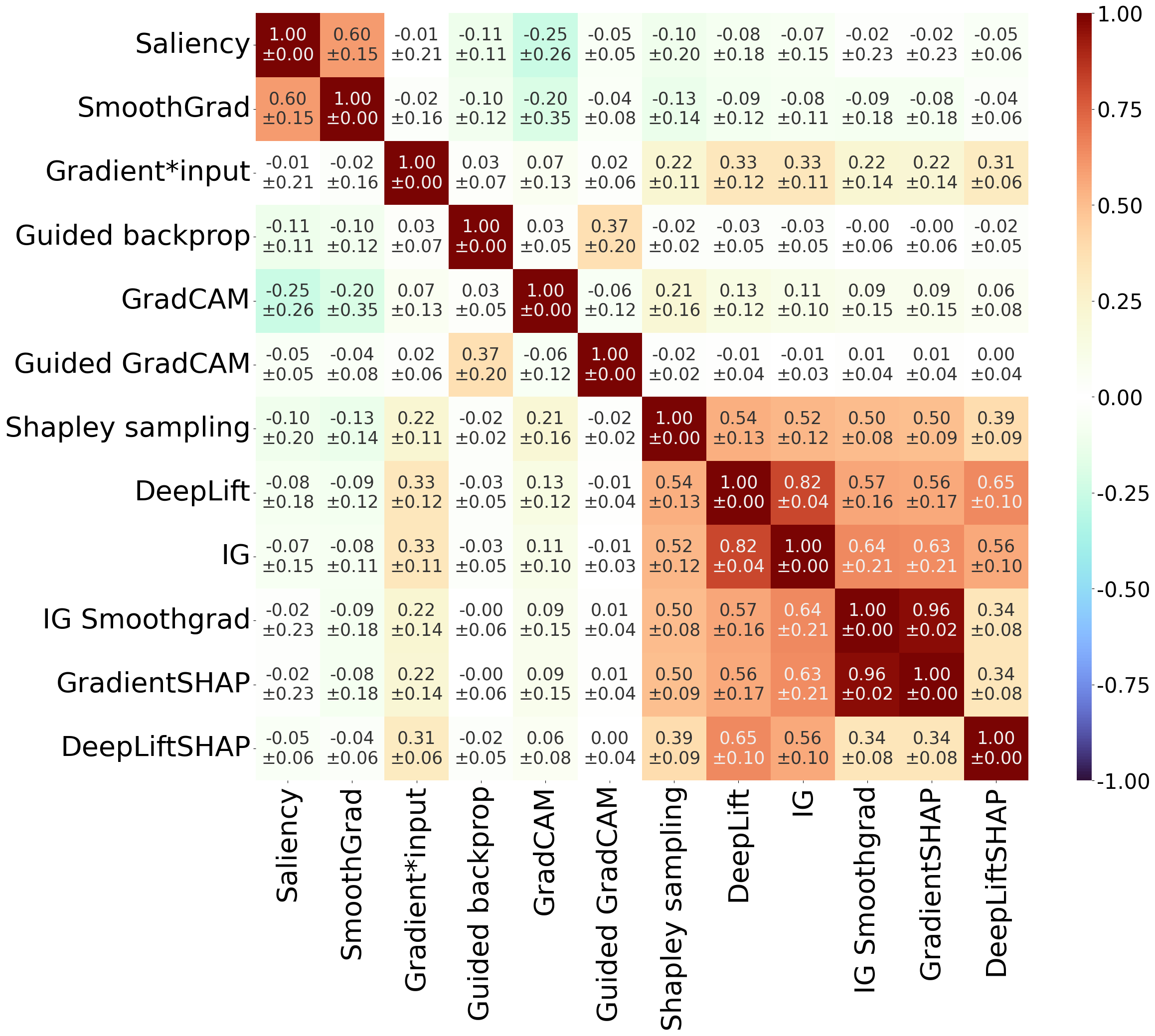
Supplementary Figure 1**. Pairwise Pearson correlations of attribution methods, including mean and standard deviation. For each test set ECG, correlations were calculated between attribution methods. The values presented here represent the average correlations across the test set and the nine ECG classes. *Abbreviations*. IG, Integrated gradients.


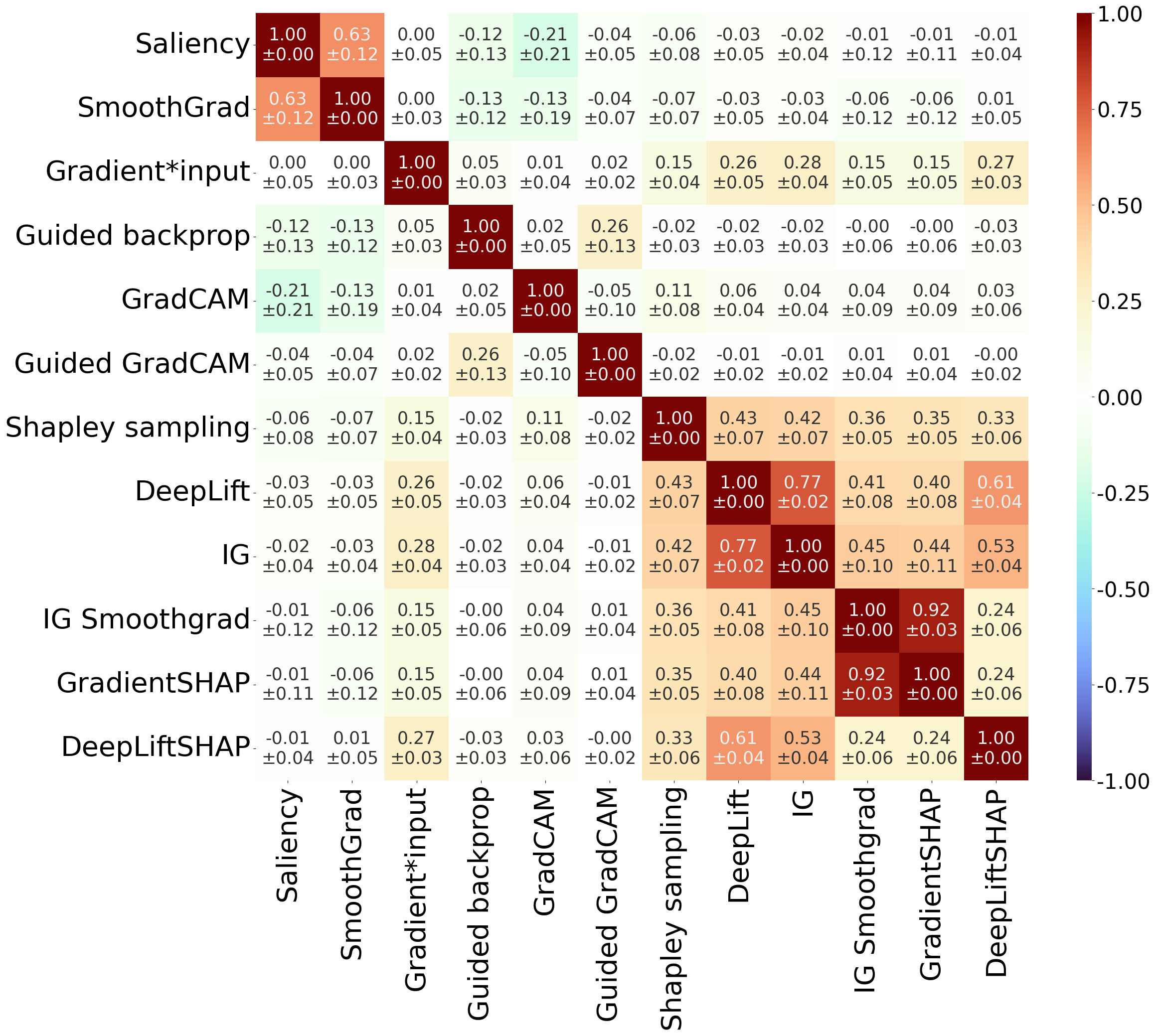


**Supplementary Figure 2**. Pairwise Spearman correlations of attribution methods, including mean and standard deviation. For each test set ECG, correlations were calculated between attribution methods. The values presented here represent the average correlations across the test set and the nine ECG classes. *Abbreviations*. IG, Integrated gradients.


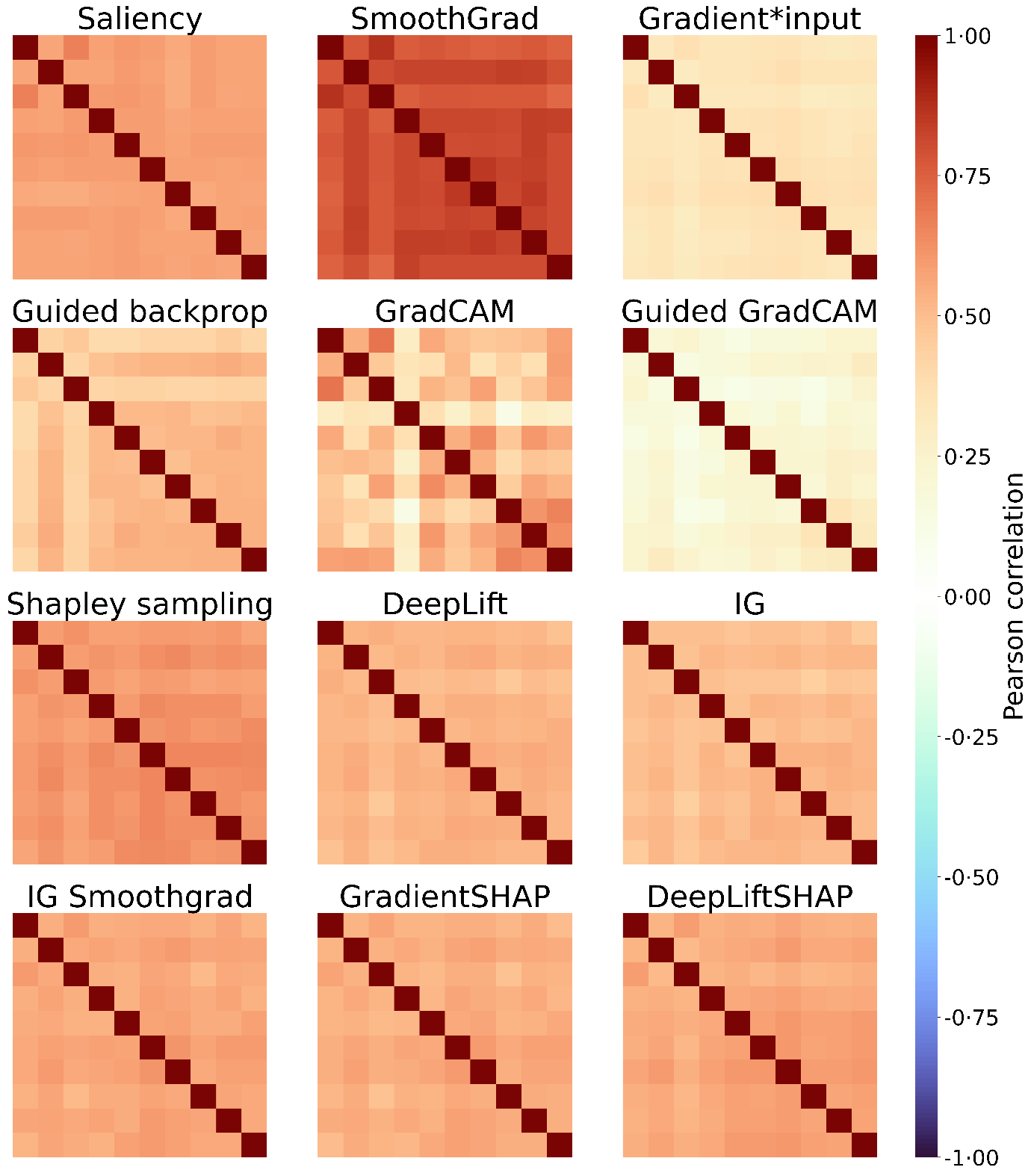
**Supplementary Figure 3**. Pairwise Pearson correlations of attribution methods across model seeds. For each test set ECG, correlations were calculated between model seeds. The values presented here represent the average correlations across the test set and the nine ECG classes. *Abbreviations*. IG, Integrated gradients; Guided backprop; Guided backpropagation.


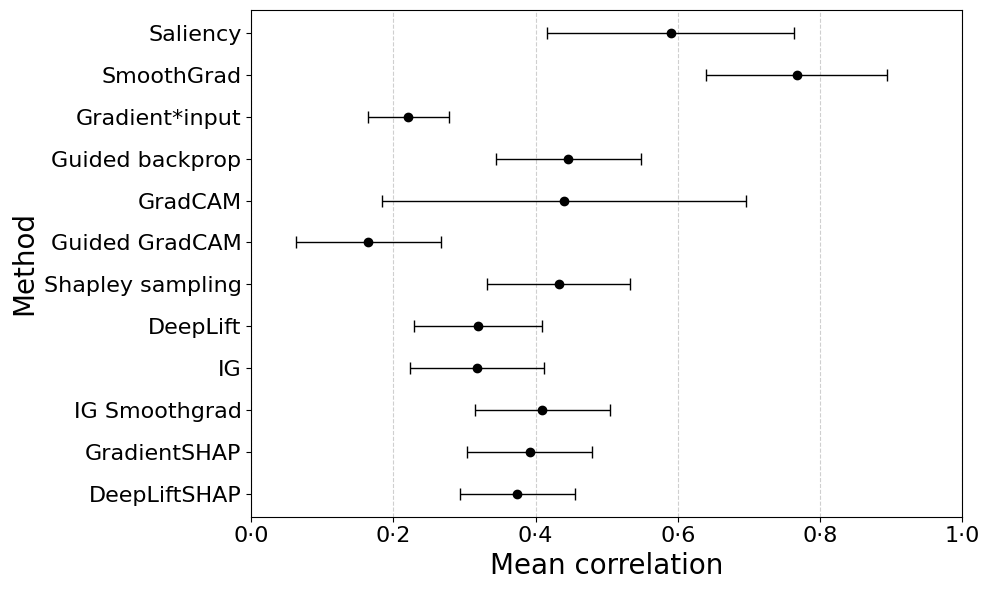


**Supplementary Figure 4**. Self-consistency: Spearman correlations between attribution maps across seeds produced by the same attribution method, averaged over ten seeds and nine classes. Abbreviations. IG, Integrated gradients.


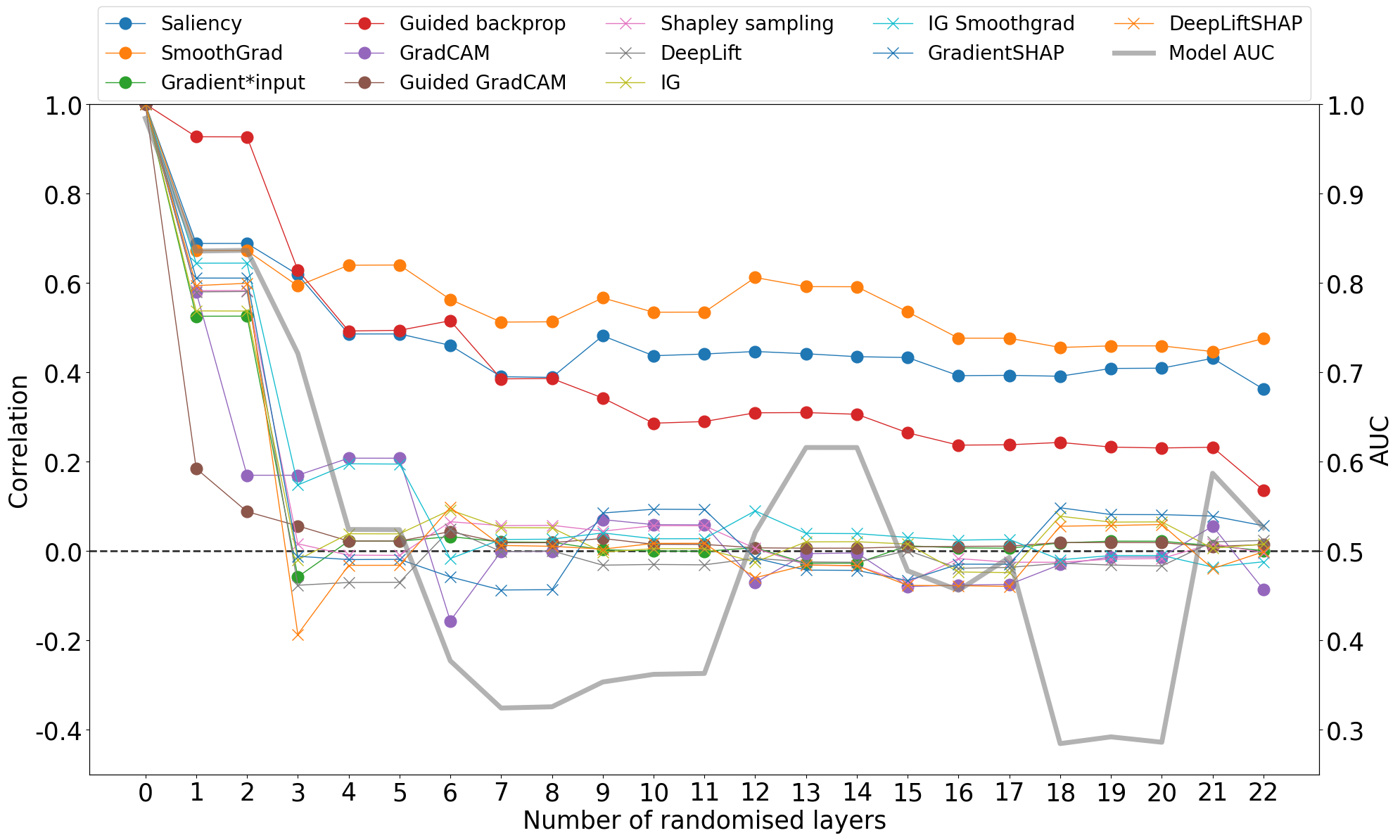


**Supplementary Figure 5**. Dependence on model weights: progressive randomisation of a model trained to detect atrial fibrillation. The y-axis shows the Spearman correlation between the attribution maps produced using the non-randomised model and each randomisation step. The x-axis shows the amount of randomised convolutional layers, starting at the output layer of the network.

# Supplementary tables

**Supplementary Table 1**. Model architecture.

| **Block / layer** | **Layer composition** | **Pooling** | **In channels** | **Out channels** | **Kernel / stride** | **Dropout** |
| --- | --- | --- | --- | --- | --- | --- |
| First convolution | BN, Pad, Conv, ReLU, BN | - | 8 | 16 | k=7, s=1 | - |
| Residual block 1 | BN, ReLU, Pad, Conv, ReLU, BN, Dropout, Pad, Conv, BN | MaxPool (k=1, s=1) | 16 | 16 | k=7, s=1  k=7, s=1 | 0.1 |
| Residual block 2 | BN, ReLU, Pad, Conv, ReLU, BN, Dropout, Pad, Conv, BN | MaxPool (k=1, s=1) | 16 | 32 | k=7, s=1  k=7, s=1 | 0.1 |
| Residual block 3 | BN, ReLU, Pad, Conv, ReLU, BN, Dropout, Pad, Conv, BN | MaxPool (k=2, s=2) | 32 | 32 | k=7, s=2  k=7, s=1 | 0.1 |
| Residual block 4 | BN, ReLU, Pad, Conv, ReLU, BN, Dropout, Pad, Conv, BN | MaxPool (k=1, s=1) | 32 | 64 | k=7, s=1  k=7, s=1 | 0.1 |
| Residual block 5 | BN, ReLU, Pad, Conv, ReLU, BN, Dropout, Pad, Conv, BN | MaxPool (k=2, s=2) | 64 | 64 | k=7, s=2  k=7, s=1 | 0.1 |
| Residual block 6 | BN, ReLU, Pad, Conv, ReLU, BN, Dropout, Pad, Conv, BN | MaxPool (k=1, s=1) | 64 | 128 | k=7, s=1  k=7, s=1 | 0.1 |
| Residual block 7 | BN, ReLU, Pad, Conv, ReLU, BN, Dropout, Pad, Conv, BN | MaxPool (k=2, s=2) | 128 | 128 | k=7, s=2  k=7, s=1 | 0.1 |
| Flatten | Flatten | - | 128 | 9600 | - | - |
| Linear + reshape | Linear, Reshape | - | 9600 | 1 | - | - |

**Supplementary Table 2**. Binary classification performance per class on the test set. Displayed are means across seeds with standard deviation between brackets.

|  | AUROC | AUPRC | Precision | Recall | Accuracy | Micro F1 | Specificity |
| --- | --- | --- | --- | --- | --- | --- | --- |
| Sinus rhythm | 0.980 (0.001) | 0.986 (0.001) | 0.962 (0.011) | 0.910 (0.013) | 0.925 (0.002) | 0.935 (0.003) | 0.947 (0.016) |
| Atrial fibrillation | 0.985 (0.001) | 0.851 (0.012) | 0.510 (0.065) | 0.931 (0.040) | 0.934 (0.018) | 0.654 (0.048) | 0.935 (0.021) |
| Sinus bradycardia | 0.957 (0.002) | 0.640 (0.026) | 0.477 (0.034) | 0.950 (0.019) | 0.883 (0.015) | 0.634 (0.026) | 0.875 (0.019) |
| Sinus tachycardia | 0.994 (0.001) | 0.960 (0.005) | 0.819 (0.059) | 0.919 (0.031) | 0.976 (0.006) | 0.863 (0.024) | 0.981 (0.009) |
| First degree AV block | 0.994 (0.001) | 0.928 (0.010) | 0.606 (0.121) | 0.967 (0.019) | 0.953 (0.021) | 0.737 (0.085) | 0.952 (0.023) |
| Right bundle branch block | 0.996 (0.001) | 0.982 (0.002) | 0.756 (0.015) | 0.993 (0.003) | 0.942 (0.004) | 0.859 (0.009) | 0.931 (0.006) |
| Left bundle branch block | 0.992 (0.001) | 0.923 (0.007) | 0.619 (0.042) | 0.974 (0.019) | 0.946 (0.008) | 0.755 (0.026) | 0.943 (0.011) |
| Low QRS voltage | 0.994 (0.001) | 0.865 (0.031) | 0.569 (0.157) | 0.886 (0.115) | 0.974 (0.011) | 0.667 (0.083) | 0.977 (0.014) |
| Left ventricular hypertrophy | 0.980 (0.003) | 0.876 (0.008) | 0.656 (0.062) | 0.901 (0.029) | 0.955 (0.010) | 0.756 (0.036) | 0.959 (0.012) |

*Abbreviations.* AUPRC, area under the precision recall curve; AUROC, area under the receiver operator curve; AV, atrioventricular.

**Supplementary Table 3**. Pairwise Pearson correlations of attribution methods across model seeds. For each test set ECG, correlations were calculated between model seeds. The values presented here represent the average correlations across the test set and model seeds, with standard deviations between parentheses.

|  | **All** | **Sinus rhythm** | **Atrial fibrillation** | **Sinus bradycardia** | **Sinus tachycardia** | **First degree AV block** | **RBBB** | **LBBB** | **Low QRS voltage** | **LVH** |
| --- | --- | --- | --- | --- | --- | --- | --- | --- | --- | --- |
| Saliency | 0.58 (0.11) | 0.64 (0.02) | 0.61 (0.03) | 0.67 (0.03) | 0.46 (0.05) | 0.72 (0.02) | 0.62 (0.03) | 0.61 (0.04) | 0.49 (0.06) | 0.42 (0.10) |
| SmoothGrad | 0.80 (0.11) | 0.73 (0.12) | 0.86 (0.09) | 0.90 (0.06) | 0.91 (0.05) | 0.83 (0.10) | 0.91 (0.07) | 0.79 (0.14) | 0.85 (0.08) | 0.77 (0.11) |
| Gradient*input | 0.35 (0.09) | 0.54 (0.20) | 0.38 (0.05) | 0.46 (0.21) | 0.43 (0.23) | 0.49 (0.21) | 0.38 (0.25) | 0.42 (0.23) | 0.35 (0.26) | 0.38 (0.25) |
| Guided backpropagation | 0.48 (0.11) | 0.50 (0.17) | 0.52 (0.17) | 0.63 (0.07) | 0.63 (0.18) | 0.50 (0.17) | 0.61 (0.15) | 0.58 (0.14) | 0.53 (0.19) | 0.45 (0.19) |
| GradCAM | 0.47 (0.38) | 0.66 (0.22) | 0.61 (0.49) | 0.38 (0.48) | 0.41 (0.31) | 0.69 (0.23) | 0.62 (0.18) | 0.28 (0.39) | 0.42 (0.33) | 0.42 (0.33) |
| Guided GradCAM | 0.22 (0.15) | 0.22 (0.13) | 0.19 (0.08) | 0.13 (0.10) | 0.20 (0.16) | 0.46 (0.11) | 0.12 (0.07) | 0.26 (0.18) | 0.12 (0.11) | 0.25 (0.09) |
| Shapley sampling | 0.61 (0.11) | 0.65 (0.03) | 0.48 (0.07) | 0.68 (0.03) | 0.78 (0.02) | 0.44 (0.08) | 0.66 (0.03) | 0.61 (0.03) | 0.68 (0.05) | 0.51 (0.04) |
| DeepLift | 0.53 (0.13) | 0.49 (0.24) | 0.62 (0.18) | 0.48 (0.25) | 0.71 (0.14) | 0.74 (0.14) | 0.49 (0.10) | 0.66 (0.17) | 0.52 (0.23) | 0.71 (0.15) |
| IG | 0.50 (0.13) | 0.68 (0.17) | 0.49 (0.21) | 0.56 (0.18) | 0.48 (0.23) | 0.65 (0.14) | 0.66 (0.19) | 0.52 (0.12) | 0.66 (0.15) | 0.53 (0.20) |
| IG Smoothgrad | 0.56 (0.11) | 0.64 (0.12) | 0.64 (0.15) | 0.58 (0.15) | 0.57 (0.16) | 0.57 (0.17) | 0.70 (0.10) | 0.60 (0.22) | 0.57 (0.09) | 0.63 (0.12) |
| GradientSHAP | 0.55 (0.11) | 0.56 (0.10) | 0.62 (0.09) | 0.56 (0.13) | 0.56 (0.12) | 0.52 (0.14) | 0.60 (0.14) | 0.69 (0.07) | 0.48 (0.21) | 0.54 (0.06) |
| DeepLiftSHAP | 0.56 (0.10) | 0.61 (0.04) | 0.46 (0.03) | 0.61 (0.07) | 0.42 (0.04) | 0.59 (0.03) | 0.48 (0.06) | 0.56 (0.05) | 0.67 (0.08) | 0.66 (0.04) |
